# Torsades de Pointes With a Normal Magnesium Level in the Setting of Short Bowel Syndrome

**DOI:** 10.7759/cureus.16743

**Published:** 2021-07-29

**Authors:** Talhah Z Siraj, Ismail Ganim, William Barker, Joseph Abraham, Eric Landa

**Affiliations:** 1 Internal Medicine, Unity Health, Searcy, USA

**Keywords:** torsades de pointes (tdp), serum magnesium, short bowel syndrome, isoproterenol, arrhythmia

## Abstract

Torsades de pointes (TdP) is a potentially fatal arrhythmia, typically presenting with a congenital or acquired etiology. Low serum magnesium level is a known cause leading to this arrhythmia. However, it has been found that even in the setting of a normal serum magnesium level and with no other foreseeable etiology, TdP may still occur, especially in those with chronic electrolyte deficiencies. TdP may be treated in a number of ways, including IV magnesium sulfate or defibrillation if the patient becomes unresponsive and hemodynamically unstable. In some cases, atrial overdrive is required with the use of isoproterenol. A final decision, however, would necessitate asking if the patient can be sent home on medical management to prevent recurrence of the arrhythmia or require placement of a permanent pacemaker. Here, we describe a patient developing recurrent TdP despite normal serum magnesium level in the setting of short bowel syndrome.

## Introduction

Torsades de pointes (TdP) is a type of polymorphic ventricular tachycardia characterized by a gradual change in amplitude and twisting of the QRS complex around an isoelectric line typically noted on an electrocardiogram [1]. This arrhythmia is commonly associated with a prolonged QTc, which is a heart rate-adjusted prolongation of the QT interval. The QT interval is considered long when greater than 460 milliseconds (ms) in females and 450 ms in males. If the QT interval is greater than 500 ms, then there is a two- to three-fold increased risk of developing TdP. Prolonged QT syndrome is subdivided into congenital or acquired. Congenital prolonged QT syndrome is associated with mutations in LQTS1, LQTS2, and LQT3, with a majority of congenital causes occurring due to mutations with LQTS1 [2]. Acquired causes can range from drug-induced to low magnesium levels as the etiology of the prolongation. Some of the more common drug classes that may cause QT prolongation include Class Ia and III antiarrhythmics, antipsychotics, antihistamines, and some antibiotics, such as fluoroquinolones and macrolides among many others. Low magnesium levels may potentiate prolonged QT syndrome as well. Hypomagnesemia is generally caused by inadequate magnesium intake or impairment of renal conservation or gastrointestinal absorption [3]. The treatment of TdP that develops out of QT prolongation may vary. IV or oral magnesium has been shown to have some benefits in hemodynamically stable patients who have drug-induced TdP. If the patient is hemodynamically unstable or in cardiac arrest due to TdP, electrical cardioversion is recommended. Synchronized cardioversion should be performed if the patient is hemodynamically unstable with a pulse. If the patient lacks a pulse, then defibrillation is necessary. Pharmacologically, it has been shown that isoproterenol, a non-selective beta-agonist, can increase the heart rate and shorten the QT interval of a patient, thereby preventing further episodes of TdP [1]. Normally given after the failure of treatment with magnesium, isoproterenol has also been shown to terminate TdP in congenital long QT syndrome and antipsychotic-induced TdP [4,5]. Here, we present a case of a patient who developed recurrent episodes of TdP in the setting of a normal magnesium level with an extensively altered gastrointestinal anatomy.

## Case presentation

An 89-year-old male presented from a nursing home with potential seizure-like activity witnessed by his daughter. He had an extensive medical history that included colon cancer status post colectomy with ileostomy placement and significant resection of the small bowel secondary to small bowel obstruction caused by adhesions. Furthermore, he suffered from malnutrition and chronic electrolyte abnormalities, given his short bowel syndrome secondary to multiple resections. 

**Figure 1:**
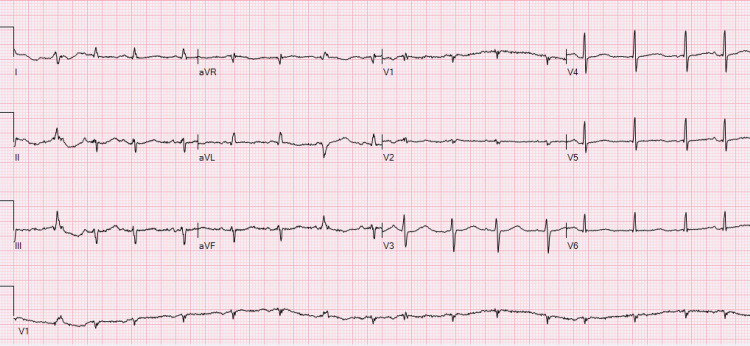
Initial QT prolongation This 12-lead EKG shows QT prolongation of 590 ms, which is an acute change from the patient’s most recent EKG prior to this admission.

The daughter noticed her father became unresponsive hence initiated bedside cardiopulmonary resuscitation (CPR) for two minutes until emergency medical services (EMS) arrived. Upon arrival to the emergency department (ED), the patient was noted to have a serum magnesium level of 0.6 mg/dl, and an EKG revealed a prolonged QTc of 590 ms, as noted in Figure 1.

Two grams of magnesium sulfate IV push was administered, and the patient was then transferred to the intensive care unit (ICU). Later that afternoon, the patient’s QTc began to normalize, and his magnesium was then 2.5 mg/dl. Given that he appeared medically stable and had no capacity issues at the hospital, he was placed back in the ED for ICU holding. 

**Figure 2:**
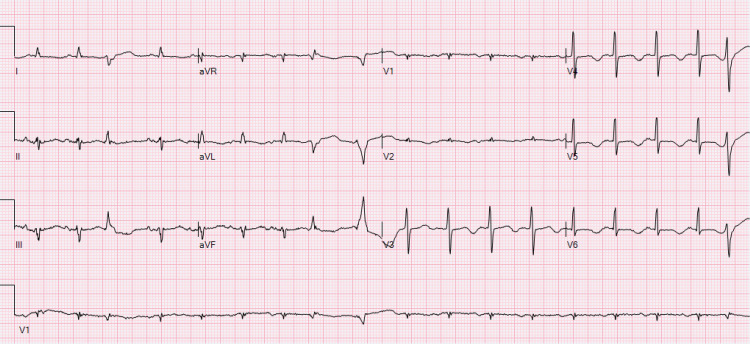
Recurrent QT prolongation after initial correction Twelve-lead EKG showing recurrence of QT prolongation to 573 ms after initial improvement overnight. The patient would continue to develop recurrent episodes of TdP. TdP: torsades de pointes.

The following morning, the patient began having intermittent episodes of TdP as noted on telemetry. His QTc prolonged again to 573 ms (Figure 2); however, his serum magnesium remained normal.

**Figure 3:**
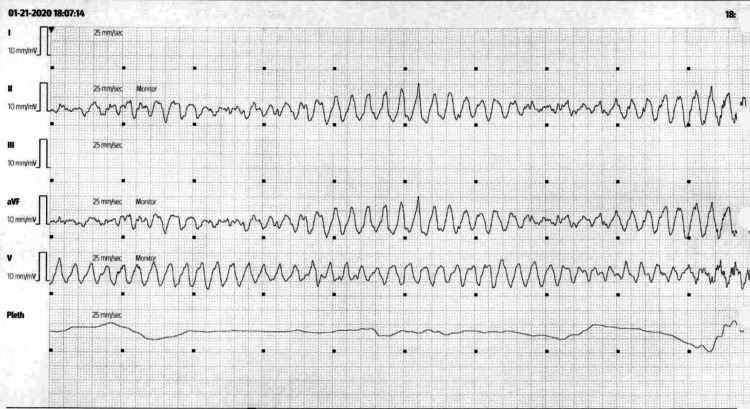
Telemetry strip of the patient developing TdP Image of the moment the patient developed his final TdP rhythm requiring defibrillation. The patient developed several short non-sustained runs of TdP in the 20 minutes prior to this episode. TdP: torsades de pointes.

He was given additional magnesium along with IV metoprolol and two bolus doses of amiodarone. He required defibrillation for these episodes of TdP each time, at which point cardiology was consulted. The episodes of TdP were successfully terminated each time, and magnesium level never dropped below normal. Cardiology recommended using isoproterenol if episodes of TdP were to recur. He remained stable throughout the day until later that evening, when the patient became unresponsive and lost his pulse as he developed another episode of TdP witnessed by the ICU team (Figure 3).

**Figure 4:**
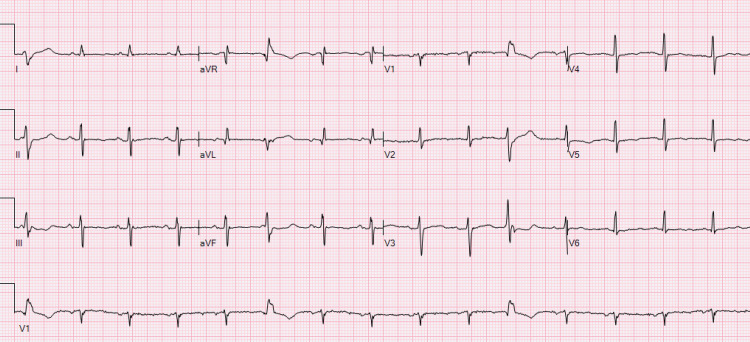
Twelve-lead EKG following PPM placement This final 12-lead EKG shows a complete resolution of the patient’s QT interval to 406 ms after the placement of the PPM set at a higher rate. PPM: permanent pacemaker.

Immediate defibrillation was successful in attaining a normal sinus rhythm, and isoproterenol was initiated for the following 24 hours. As per cardiology, we maintained the patient’s potassium above 4 mg/dl and magnesium above 2 mg/dl, which required recurrent replacement throughout admission. The patient did not develop any arrhythmias on isoproterenol and appeared stable once removed from it after the 24 hours was complete. Around midnight, the patient developed another brief episode of TdP; however, it spontaneously converted back to sinus rhythm and did not require defibrillation. Isoproterenol was added back at half dose, and the following morning, cardiology recommended for permanent pacemaker (PPM) placement in the management of persistent TdP. The patient agreed and had a successful placement of his pacemaker the following morning with a higher rate setting, given his propensity to develop arrhythmia. Following pacemaker placement, the patient no longer developed episodes of TdP. With exception to when he first arrived in the ED, his magnesium level never dropped below 1.8 mg/dl and mostly stayed above 2 mg/dl. His QTc normalized to 406 ms following PPM placement (Figure 4).

The patient was stable for transfer out of the ICU to the telemetry floor. He did not develop any arrhythmias the rest of admission and was then discharged back to the nursing home in stable condition six days later.

## Discussion

TdP can be a detrimental arrhythmia that, if not treated immediately, can lead to mortality. The first step is to determine the etiology. TdP is almost always preceded by QT prolongation, normally above 500 ms. Prolongation of the QT interval can be congenital or acquired. When considering an acquired cause of TdP, the most common is drug-induced or hypomagnesemia. When a patient presents with TdP to the ED, a serum magnesium level is commonly collected and often found to be low. Serum magnesium level may be an inaccurate measure for body magnesium stores, particularly for those with chronic deficiencies [6]. A study has shown that magnesium deficiency is associated with an increased prevalence and risk of 11 major conditions [7]. It additionally noted that magnesium deficiency could predict adverse events and found a decreased chance of pathology if supplementation was instituted despite a normal serum magnesium level.

The patient presented in this case had chronic electrolyte deficiencies secondary to multiple small and large bowel resections. This included the terminal ileum, which is responsible for magnesium absorption. Following these resections, the patient suffered from short bowel syndrome where he had less than 180 cm of bowel remaining for appropriate solute and water absorption [8]. The patient had chronic vitamin and electrolyte deficiencies and was on several oral supplements, including magnesium oxide. Despite this, he still presented with a magnesium level of 0.6 mg/dl. Knowing how magnesium stores may be low despite a normal serum magnesium level, the fact that this patient’s level was also low was worrisome. His significant hypomagnesemia secondary to short bowel syndrome potentiated recurrent episodes of TdP upon presentation to the ED. Initially, he had a low magnesium level. However, despite receiving appropriate magnesium supplementation and achieving a normal serum magnesium level, he continued to develop TdP. This indicated that his body stores of magnesium continued to remain low and that the supplementation was not appropriately absorbed due to his short bowel syndrome. 

The continuation of the patient’s arrhythmias caused a problem that required a permanent fix. Isoproterenol has been proven to treat TdP by increasing the heart rate to a level where the risk of recurrent arrhythmias is low. However, this is only a temporizing measure. Plus, removal of the isoproterenol can cause the arrhythmias to recur, as evident in this patient. Therefore, the most appropriate correction for the arrhythmia was a pacemaker. The pacemaker needs to be permanently adjusted at a higher ventricular rate, typically between 90 and 110 beats per minute (bpm), to overcome the arrhythmia [1]. Other etiologies for non-recurrent TdP can be treated with appropriate IV magnesium supplementation along with potassium and calcium supplementation. Discontinuation of any medications that may cause a prolonged QT interval is prudent for these patients as well [1]. 

## Conclusions

We presented a case of an elderly man who had severe short bowel syndrome and chronic electrolyte deficiencies for many years. Despite being on severe oral supplements, including magnesium oxide, the patient still presented with a very low magnesium level that ultimately led to his TdP episodes even when the measured serum magnesium level was normal. Isoproterenol was temporarily used to terminate the arrhythmia until a PPM was placed. This observation helped us to understand that without any other known etiology, a chronically depleted body magnesium store in a patient with short bowel syndrome may lead to the development of fatal arrhythmias such as TdP.
